# Serving Patterns of Women’s Badminton Medalists in the Rio 2016 Olympic Games

**DOI:** 10.3389/fpsyg.2020.00136

**Published:** 2020-02-05

**Authors:** Miguel-Ángel Gómez-Ruano, Adrián Cid, Fernando Rivas, Luis-Miguel Ruiz

**Affiliations:** ^1^Faculty of Physical Activity and Sports Sciences, Technical University of Madrid, Madrid, Spain; ^2^Faculty of Education, University of Vigo, Vigo, Spain; ^3^Spanish Badminton Federation, Madrid, Spain

**Keywords:** racket sports, performance indicators, female, notational analysis, elite player performance

## Abstract

The aim of the present study was to describe and identify the serving performance profiles of medalists during an elite women’s badminton tournament taking notational and temporal variables into account. The sample was composed of the 14 matches (*n* = 1,052 rallies) played by the three medalists during the 2016 women’s singles Olympic Games badminton event (Rio, Brazil). The independent variable studied was serving player (medalist/opponent); while the dependent variables were related to notational analysis: serve type, set, and point won by the server/receiver; and the time-related variables: number of strokes per rally, rally time, rest time, and frequency of strokes. The main results showed that: (i) temporal parameters were similar for total match duration but shorter for rally time, and longer for rest time and with more strokes per rally than found in previous research; (ii) the serve effectiveness showed neutral values when analyzing serving by all the players, medalists, and opponents (around 50%); (iii) the two-step cluster analysis identified how successful players used the serve when playing short rallies with backhand short and flick serves (cluster 1), and forehand long serves (cluster 2); and during long rallies with the use of the backhand short serve, forehand short serve and forehand long serve (cluster 3). On the other hand, medalists and their opponents used forehand long serves during set 1 with durations of 8.80 s (cluster 5); and the opponents showed an independent performance using the forehand short serve during sets 1 and 2 (cluster 4); and (iv) the classification tree analysis (Exhaustive CHAID) identified the importance of different serving patterns with the gold medal player using more backhand and forehand flick serves, and the main use of backhand short serves during sets 1 and 2 in all the tournament stages. The bronze medalist used more forehand long serves during all sets, and the silver medalist showed a mixed performance of serves using the forehand short serve, the backhand short serve and the forehand long serve. The current findings may help coaches and players to manage different serving and playing patterns during training and matches according to the serve and rally requirements.

## Introduction

Badminton is a sport characterized by a combination of speed, endurance, and power displayed during high intensity and short-duration actions that have short rest intervals between points (Laffaye et al., 2015). In particular, it is complex and dynamic and the player tries to produce quick responses (i.e., decision-making) disrupting his/her opponent’s actions, and then, win the point (Chow et al., 2014). In fact, the demands placed on a badminton player are focused on tactical, technical, and temporal adaptations to the dynamics of each context (e.g., behaviors and tactics when serving or receiving, set intervals before and after point 11, playing long or short rallies, the different importance of sets 1, 2 and 3, etc.) during the match (Abián et al., 2014; Laffaye et al., 2015). Under the current badminton regulations (i.e., scoring system) the players play more aggressively using different tactics and a higher frequency of strokes during longer matches (e.g., faster game play with more points to be played, greater variations of rally time and rest time, and more unpredictability during the intervals and sets) (Laffaye et al., 2015).

Based on this rationale, the available research on badminton (Cabello et al., 2004; Abián et al., 2014; Laffaye et al., 2015) has focused its attention on notational analysis and the temporal structure during elite competitions. On the one hand, notational analysis has been widely used to investigate the individual’s performance in badminton and racket sports providing relevant information about players’ technical and tactical behaviors during matches and rallies such as type of serve, serve effectiveness, point outcome, number of strokes used, type of shots, effectiveness when serving or receiving, etc. (Lees, 2003; Abdullahi and Coetzee, 2017). On the other hand, the temporal structure of this sport has complemented the notational analysis with relevant information about the game/match duration, rally time, rest time, density of play, number of strokes per rally or time between strokes (Phomsoupha and Laffaye, 2015; Laffaye et al., 2015). This information is extremely important due to its high applicability to real contexts when training and playing matches, setting the appropriate loads or task constraints according to the requirements of competition (Chiminazzo et al., 2018).

Specifically, these performance analyses in elite badminton have extensively studied differences according to the sex of the players, the stage or phase of competition (e.g., group or knockout stages), the final outcome of the match (i.e., winning and losing), or the players quality/strength such as the best or worst players (Barreira et al., 2016; Chiminazzo et al., 2018). However, the analysis of how successful players (i.e., medalists) perform and score points when serving at the elite level in badminton is still inconclusive. This approach has been largely studied from the perspective of developing sporting talent, analyzing the athlete (e.g., anthropometric and physiological factors, genetics, birthdate, motivation, or psychological skills), the environment (e.g., birthplace, parents, family, or coaches support), the importance of practice or training (e.g., early specialization or the volume of training), and other potential factors (e.g., injuries, recovery, or socio-economic status) of medalists (Sarkar et al., 2015; Rees et al., 2016). These characteristics showed by successful athletes reflect a determined focus when training and competing (i.e., mastering key technical, tactical, and psychological skills) with a direct impact on their performance (Starkes and Ericsson, 2003). Despite this approach of scientific research, specific performance analysis (i.e., technical, tactical, or temporal) of medalists has been developed in individual sports such as running or swimming events (Hollings et al., 2014; Mytton et al., 2015) with concluding remarks of performance features during competition that characterize their success (e.g., better performances for medalists during the last part of running or swimming races or better adaptation to different paces according to race contexts). In racket sports successful players use different effective serving and playing patterns that make it possible to defeat their opponents during rallies and matches (Chiminazzo et al., 2018).

In particular, the serve in badminton is the first stroke of the point and plays a key tactical role as it is not affected by any previous action by the opponent. The serve is thus one of the most used strokes in badminton (Abdullahi and Coetzee, 2017; Chiminazzo et al., 2018) that needs be under the full control of the server in order to potentially gain any spatial and temporal (e.g., short and long serves) advantage over the receiver during the consecutive strokes played in each point (Pearce, 2002; Alcock and Cable, 2009). However, according to Bialik (2016) the serve does not represent an advantage in women’s badminton singles where only 55% of the points were won when serving. The serve can then be considered as a way to start to play the point but not a key stroke to win direct points. Therefore, the analysis of actions performed by successful players monitoring serve type, serve effectiveness and playing patterns during the rallies may reflect their individual performance features that lead to success. Thus, the specific study of key performance indicators in elite badminton may define the characteristics of successful players when serving, and then reflect the performance profiles during their matches according to some key notational (i.e., type of serve or serve effectiveness) and temporal (i.e., number of strokes, rally time, rest time, or frequency of strokes) variables. Therefore, the aim of the present study was to describe and identify the serving performance profiles of successful players (medalists) during an elite women’s badminton tournament taking notational and temporal variables into account. It was hypothesized that successful players use different serving and playing patterns that imply quicker and more difficult technical-tactical actions to score points during the matches.

## Materials and Methods

### Sample

The sample was composed of 14 matches (Group stage, Quarter-final, Semi-finals, and Final matches) played by the three medalists (Gold, Silver, and Bronze) from the 2016 women’s singles Olympic Games badminton event (Rio, Brazil). Only one match was excluded from the sample (Bronze medal match) due to the fact that one player was injured and did not play the match. The final sample included the analysis of 1,052 rallies played by the three medalists. All matches were publicly available on TV and the data was used with the approval of the Universidad Politécnica de Madrid Ethics Committee and in accordance with the European Data Protection Law.

### Procedure

The analyses were carried out using an observation tool in a video analysis program (Dartfish, Friburgo, Switzerland). Four trained observers (graduates in Sports Sciences with 10 years’ experience as badminton coaches) collected the variables with good and very good inter and intra-rater reliability values (Kappa: >0.81; correlation coefficient *r* > 0.86; ICC: > 0.85, and standard error of measurement: < 0.46) (Altman, 1991; Hopkins, 2000).

The independent variable studied was the serving player (medalist/opponent); while the dependent variables were related to *notational analysis*: serve type (forehand short serve, forehand long serve, forehand flick, backhand short serve, and backhand flick), set (1^*st*^, 2^*nd*^, or 3^*rd*^), and point won by the server or the receiver; and the *temporal structure* variables: number of strokes per rally, rally time (time in s of the rally duration between the serve and the end of the point), rest time (time in s between the end of the point and the serve action of the next immediate point), and frequency of strokes (the time in s between opposing players’ strokes).

### Statistical Analysis

Firstly, descriptive analyses (median and lower/upper quartiles) were run for temporal parameters (total match and set duration, number of strokes per rally, rally time, rest time, and frequency of strokes) during all matches and each set (1^st^, 2^nd^, and 3^rd^) in order to show the measures of centrality of time-related demands during the championship.

Secondly, the crosstabs commands were used to study the relationships (Pearson’s Chi-square test) between the point won when serving or receiving and the type of serve used by the server (medalist or opponent). Fisher’s exact test was applied when the Expected Frequency Distribution was lower than 5 or the count of cases in one cell was lower or equal to 5 (Field, 2013). In order to estimate Effect sizes (ES) Cramer’s V test was used considering the following range values: 0.10 = small effect, 0.30 = medium effect, and 0.50 = large effect (Volker, 2006).

Thirdly, in order to analyze the variables that best explain the players’ performance when serving, the sample was grouped into different clusters that described the specificities of rallies played by the medalists and their opponents during the tournament. Then, a two-step cluster analysis was run considering the variables: type of serve, set, serving player, if the point was won by the server or the receiver, and temporal parameters (rally time, rest time, number of strokes, and frequency of strokes). The clustering technique automatically (log-likelihood distance measure) determined the best number of clusters (types of rallies played) using the Schwartz’s Bayesian Information Criterion (BIC). The model obtained was good with a Silhouette measure value of 0.5. Additionally, the clusters were differentiated using the Kruskal–Wallis H non-parametric test for numerical variables (temporal parameters: rally time, rest time, number of strokes, and frequency of strokes). The *post hoc* pairwise comparisons (Dunn’s test with the Bonferroni’s correction) were run to identify differences among clusters. The crosstabs command (Pearson’s Chi-square test) was used to differentiate the categorical variables (type of serve, set, medalist condition, and point won) among clusters.

**FIGURE 1:**
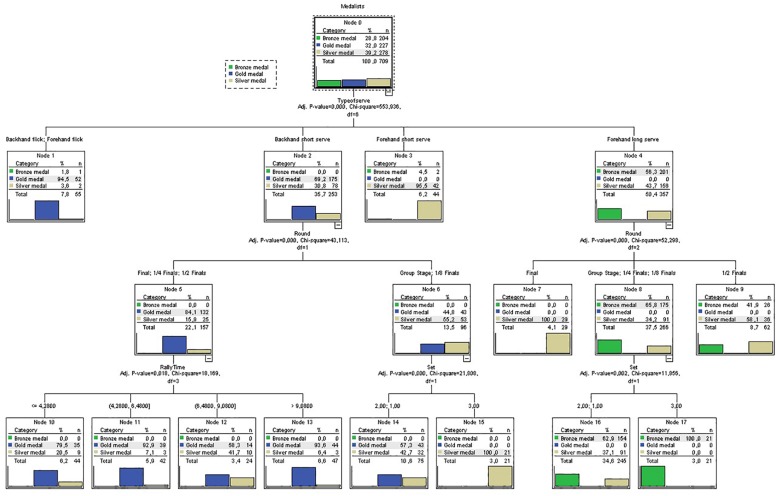
Classification tree analysis (Exhaustive CHAID) of medalists’ serving performances.

Lastly, the Exhaustive CHAID (Chi-squared automatic interaction detection) classification tree analysis was used to determine the differences between the performance playing patterns of the three medalists according to the temporal (rally time, rest time, number of strokes, and frequency of strokes) and notational (type of serve, set, set interval, round, and point won by the server, or receiver) variables. This model made it possible to split the medalists’ sample according to nodes (sub-groups) based on the impact of the medalist (gold-, silver-, or bronze-medal) condition. The algorithm used considers a nominal dependent variable and nominal and numerical independent variables. The Chi-square test identifies the relationships between independent variables, and then finds the best predictors (temporal and notational variables) that most influence the dependent variable (Schnell et al., 2013). The algorithm used completes three steps on each node of the root (merging, splitting, and stopping) in order to find the predictors that exert the most influence on the dependent variable. The exhaustive CHAID assesses all splitting possibilities for each independent variable, and the merging step improves the searching procedure to find (and merge) those similar pairs until only a single pair remains. The model provides a graphical presentation of the final tree (hierarchical tree, see Figure 1) where the impact of each independent variable makes it possible to split the root node (node 0) into branches with *n* descendent nodes. The tree continues descending with each branch that assesses the remaining significant independent variables (improving the search of splitting nodes). The terminal nodes are established when no further split can be made (Schnell et al., 2013).

The statistical specifications considered in this model were: (i) *p* < 0.05; (ii) Pearson’s Chi-square test was used to check relationships among independent variables; (iii) the maximum number of iterations was 100; (iv) the minimum change in expected cell frequencies was 0.001; (v) the Bonferroni adjustment was used; and (vi) a maximum of three levels were considered in the tree model. Lastly, the risk of misclassification was estimated as a measure of model reliability (Schnell et al., 2013). All statistical analyses were performed using the statistical software IBM SPSS statistics for Windows, version 22.0 (IBM, Corp., Armonk, NY, United States).

## Results

Table 1 shows the descriptive results of temporal variables (median, lower, and upper quartile) during the matches played by medalists during the Tournament.

**TABLE 1:** Descriptive results (median, lower, and upper quartile) for match and set temporal parameters during the matches studied.

		Quartile	
		
	Median	Lower	Upper
Match duration (min)	41.8	38.3	53.5
Set 1 duration (min)	22.1	17.7	24.8
Set 2 duration (min)	20.7	17.9	23.3
Set 3 duration (min)	41.8	38.3	53.5
Rally time (s)	7.87	4.74	12.6
Rest time (s)	22.1	17.1	29.6
Strokes per rally (n)	8.0	5.0	13.0
Frequency (s)	1.01	0.90	1.12
**Set 1**			
Rally time (s)	7.78	4.93	12.6
Rest time (s)	21.7	17.0	29.7
Strokes per rally (n)	7.50	5.0	13.0
Frequency (s)	1.02	0.91	1.14
**Set 2**			
Rally time (s)	7.83	4.37	12.5
Rest time (s)	21.6	16.6	29.3
Strokes per rally (n)	8.00	4.0	13.0
Frequency (s)	1.01	0.90	1.12
**Set 3**			
Rally time (s)	8.34	5.07	15.0
Rest time (s)	26.0	20.5	37.1
Strokes per rally (n)	9.0	5.0	15.0
Frequency (s)	0.97	0.88	1.07

The distribution of type of serve for points won by the server or receiver is presented in Table 2 (percentage and case numbers). The results showed that 49.6% of the points were won by the server and the type of serve was not significantly (*p* > 0.05) associated to winning the point when serving. The analysis splitting by medalist and opponents (see Table 2) showed a significant relationship for medalists between type of serve and points won when they serve with the backhand flick and backhand short serve (AR = 4.3 and 5.1, respectively). In addition, the results for opponents (see Table 2) showed significant relationships between type of serve and winning the point serving when using the forehand flick and forehand short serve (AR = 3.0 and 8.2, respectively).

**TABLE 2:** Frequency distribution of type of serve and point won by the server or receiver for all players, medalists, and opponents (Crosstab Command: Pearson’s Chi-square, degrees of freedom, significance, and effect size).

	Point won									
	
	Server			Receiver						
		
All players	*N*	%	AR	*N*	%	AR	X^2^	df	*p*	ES
Backhand flick	29	5.6	0.8	24	4.5	–0.8				
Backhand short serve	149	28.5	0.6	143	27.0	–0.6	1.284	4	0.86	0.04
Forehand flick	7	1.3	–0.2	8	1.5	0.2				
Forehand long serve	257	49.2	–0.9	276	52.1	0.9				
Forehand short serve	80	15.3	0.2	79	14.9	–0.2				
Total	522	49.6		530	50.4					
**Medalists serving**										
Backhand flick	29	8.1	4.3	1	0.4	–4.3				
Backhand short serve	131	36.7	5.1	43	17.6	–5.1	93.19†	4	< 0.001*	0.38
Forehand flick	0	0.0	–3.2	7	2.9	3.2				
Forehand long serve	178	49.9	–1.1	133	54.3	1.1				
Forehand short serve	19	5.3	–7.0	61	24.9	7.0				
Total	350	49.4		359	50.6					
**Opponents serving**										
Backhand flick	0	0.0	–3.7	23	8.1	3.7				
Backhand short serve	18	10.9	–5.6	100	35.1	5.6	105.83†	4	< 0.001*	0.48
Forehand flick	7	4.2	3.0	1	0.4	–3.0				
Forehand long serve	79	47.9	–0.5	143	50.0	0.5				
Forehand short serve	61	37.0	8.2	18	6.3	–8.2				
Total	172	50.1		171	49.9					

*p < 0.05; _†_Fisher’s exact test was used as the Expected Frequency Distribution was lower than 5. AR, adjusted residuals.

The clustering technique (two-step cluster, see Table 3) identified five different clusters (rallies) according to the notational (type of serve, set, serving player, point won by the server, or receiver) and temporal (rally time, rest time, number of strokes, and frequency of strokes) variables. The most frequent rally was cluster 3 (27.3%, played mainly by the medalists using the backhand short serve, and forehand long and short serves, during sets 2 and 3, rally duration of 12.3 s, frequency of strokes of 0.98, and greater effectiveness for the sever: 56.9%), cluster 4 (22.3%, played by the opponents using the forehand flick and forehand short serve, and backhand short serve, during sets 1 and 2, with rally times of 6.63 s, frequency of strokes of 0.92, and neutral effectiveness for the server: 51.0%), cluster 5 (21.5%, played by both players using the forehand long serve, during set 1, with rally times of 8.80 s, frequency of strokes of 1.10, and 49.0% of effectiveness for the server), cluster 1 (15.2%, played by the medalists using the backhand short serve, backhand flick and forehand short serve, during sets 1 and 2, rally times of 6.27 s, frequencies of strokes of 0.92, and 49.5% of effectiveness for the server), and cluster 2 (13.7%, played mainly by medalists using all serves (except the backhand flick), during set 2, with rally durations of 7.87 s, frequencies of strokes of 1.11, and lower effectiveness for the server: 44.7%).

**TABLE 3:** Results of rally types (clusters,% and n) identified by the two-step cluster analysis based on type of serve, serving player, set, point won by the server or receiver, rally time, rest time, frequency and number of strokes (I = predictor’s importance; and BIC = Schwartz’s Bayesian Information Criterion; Q1 = lower quartile; Q3 = upper quartile).

	Cluster 1			Cluster 2			Cluster 3			Cluster 4			Cluster 5		
					
Variables	15.2% (*n* = 160)			13.7% (*n* = 144)			27.3% (*n* = 287)			22.3% (*n* = 235)			21.5% (*n* = 226)		
					
Type of serve I = 1.0	%			%			%			%			%		
Backhand flick	17.6			0.0			1.4			0.0			0.0		
Backhand short serve	68.2			3.7			34.7			25.5			0.0		
Forehand flick	0.3			0.9			0.0			8.3			0.0		
Forehand long serve	0.0			94.5			49.3			0.0			100		
Forehand short serve	13.8			0.9			14.6			66.2			0.0		
**Serving player I = 0.69**															
Medalist	100			75.1			72.9			0.0			59.1		
Opponent	0.0			24.9			27.1			100			40.9		
**Set I = 0.42**															
Set 1	51.2			8.8			1.4			37.2			100		
Set 2	48.0			90.3			27.1			62.8			0.0		
Set 3	0.0			0.9			71.5			0.0			0.0		
**Point won I = 0.38**															
Server	49.5			44.7			56.9			51.0			49.0		
Receiver	50.5			55.3			43.1			49.0			51.0		

**Temporal variables**	**Median (Q1/Q3)**			**Median (Q1/Q3)**			**Median (Q1/Q3)**			**Median (Q1/Q3)**			**Median (Q1/Q3)**		

Rest time I = 0.12	22.2	17.1	28.3	19.4	15.3	29.9	27.5	21.8	37.6	22.5	18.7	28.4	20.5	16.2	27.2
Frequency I = 0.11	0.92	0.85	1.02	1.11	1.00	1.30	0.98	0.91	1.07	0.92	0.81	0.99	1.10	1.01	1.21
Rally time I = 0.11	6.27	3.83	9.73	7.87	4.98	11.2	12.3	6.45	21.6	6.63	3.83	12.4	8.80	6.02	13.5
Strokes I = 0.06	7.00	4.00	10.0	7.00	4.00	11.5	13.0	7.00	21.0	8.00	4.00	14.0	8.00	5.00	13.0
BIC	10326.95			9296.13			8472.99			7911.768			7456.60		

Significant differences were identified among clusters for type of serve, serving player, and set (*p* < 0.05; see Table 4). No significant (*p* > 0.05) relationships were identified among clusters for point won by the server of the receiver (Table 3). Additionally, the time-related variables showed significant differences among clusters for rally time, rest time, frequency of strokes, and number of strokes (all *p* < 0.01). The pairwise comparisons showed clear differences among rallies (clusters) with cluster 3 as the longest rally and clusters 1 and 4 as the shortest and quickest ones (see Table 4).

**TABLE 4:** Statistical differences among clusters in the categorical and numerical variables analyzed.

Categorical variables	χ^2^	*p*	ES (ESI)
Type of serve	1319.62†	<0.001	0.54 (Large effect)
Serving player	455.381	<0.001	0.66 (Large effect)
Set	1169.68	<0.001	0.75 (Large effect)
Point won	5.346	0.252	0.07 (Small effect)

**Numerical variables**	**χ^2^**	***p***	***Post hoc***

Rest time	53.605	<0.001	1 vs. 2-3; 2 vs. 4-5; 3 vs. 5
Frequency	258.550	<0.001	2 vs. 1-3-4-5; 5 vs. 1-3-4
Rally time	97.400	<0.001	3 vs. 1-2-4-5; 5 vs. 1-2-4
Strokes	71.933	<0.001	1 vs. 5; 2 vs. 5; 3 vs. 1-2-4-5

*_†_Fisher’s exact test was used as the Expected Frequency Distribution was lower than 5; ESI = effect size interpretation.*

The classification tree model was run to identify specific playing performances of medalists when monitoring for temporal (number of strokes, rally time, rest time, and frequency of strokes) and notational (tournament round, set, interval, type of service, and outcome) variables in the statistical analysis. The results showed only four significant variables (type of serve, round, set, and rally time) when classifying medalists’ performance (three-stage tree). The following factors led to 17 nodes (12 final nodes) of contrasting groups classifying medalists mainly by type of serve (level 1), round (level 2) and rally time and set (level 3). Figure 1 shows the categories for predictor variable (medalists: gold-, silver-, and bronze-medal) and also the 17 nodes defined by the classification tree model.

Level 1 (root node) is split by the type of serve showing the gold medalist using more backhand flicks and forehand flicks (node 1: 94.5%; *n* = 52) and backhand short serves (node 2: 69.2%; *n* = 175). The silver medalist used more forehand short serves (node 3: 95.5%; *n* = 42) and the bronze medalist used more forehand long serves (node 4: 56.4%; *n* = 201). Level 2 showed the importance of the round for the backhand short serve (from node 2) where there was a greater use of this serve during the final, semi-finals, and quarter-finals by the gold medalist (node 5: 84.1%; *n* = 132) and during the group stage and round of 16 for the silver medalist (node 6: 55.2%; *n* = 53). In addition, level 2 showed the importance of the round for the forehand long serve (from node 4) where the silver medalist used this serve more often during the final and semi-finals (nodes 7: 100%; *n* = 29; and node 9: 58.1%; *n* = 36, respectively) and the bronze medalist during the group stage, quarter-final, and round of 16 (node 8: 65.8%; *n* = 175).

Level 3 showed the importance of rally time when using the backhand short serve during the final, semi-finals and quarter-finals by the gold medalist (from node 5), the set when using the backhand short serve during group stage and round of 16 (from node 6), and the set when using the forehand long serve during the group stage, quarter-final and round of 16 (from node 8). On the one hand, the importance of rally time showed greater use of the backhand short serve by the gold medalist during rallies with time durations ranged between 4.28 and 6.48 s (node 11: 92.9%; *n* = 39) and longer than 9.08 s (node 13: 93.6%; *n* = 44). In addition, the use of the backhand short serve was greater by the gold medalist during sets 1 and 2 of the group stage, quarter-final and round of 16 (node 14: 57.3%; *n* = 43), and greater by the silver medalist during set 3 (node 15: 100%; *n* = 21). On the other hand, the significant effect of the set for the bronze medalist showed more actions when using forehand long serves during the group stage, quarter-final, and round of 16 matches in set 3 (node 17: 100%; *n* = 21) and sets 1 and 2 (node 16: 62.9%; *n* = 154) than the silver medalist. The classification tree model explained 74.8% of total variance after cross-validation analysis.

## Discussion

The aim of the current study was to describe and identify the serving performance profiles of rallies played by successful players (medalists) when taking notational and temporal variables into account during the women’s badminton Olympic Games (Rio, 2016). As was argued successful players (medalists) performed differently when playing rallies using a wider range of serve types than their opponents with a different impact on their points and match behaviors as identified in the two-step cluster and decision tree analyses (Starkes and Ericsson, 2003; Rees et al., 2016). These main findings may reflect a better technical and tactical preparation to serve and play the point managing fatigue, next point preparation or stress/pressure during the set/match (Taylor et al., 2008; Barreira et al., 2016; Chiminazzo et al., 2018).

### Temporal Analysis

The results of temporal parameters of matches played by medalists during the tournament showed similar total match duration to previous research that studied international badminton tournaments (Cabello et al., 2004; Torres-Luque et al., 2019). However, the rally time of current matches showed shorter durations (7.87 compared with values of 9–10 s) than previous studies. This finding reflects the fact that during the tournament successful players showed the same total time duration but played rallies at a higher intensity (8 strokes per rally and frequency of strokes of 1.01) and with longer rest time periods. This general trend is in agreement with Phomsoupha and Laffaye (2015) and Chiminazzo et al. (2018) who described a new temporal structure of elite badminton with high-intensity and short-duration intermittent actions that require longer rest periods. Along these lines, successful players may reflect a better adaptation to playing quick actions due to a better mastery of technical, tactical, and psychological abilities with a direct impact on match behaviors (Starkes and Ericsson, 2003). Additionally, medalists played sets 1 and 2 with similar time duration but shorter rally time, more strokes per rally, shorter frequency of strokes, and longer rest time periods than presented in the available research (Torres-Luque et al., 2019). Specifically, these results suggest a better use by medalists of stoppages, end of points and breaks for managing pressure, fatigue, and recovery than their opponents (Taylor et al., 2008). However, during set 3 medalists recorded a longer set duration, and rest time, but shorter rally time than in a previous study that analyzed the whole competition (Torres-Luque et al., 2019). This result may reflect the fact that medalists usually play the decisive set 3 only during the eliminatory phase where the highest level of performance between players generates an open outcome. Thus, successful players used stoppages, end of points and breaks for managing pressure, fatigue, and recovery during these critical moments of the match (Taylor et al., 2008).

On the other hand, the specific sample of the Olympic Games and the analysis of only medalists’ matches may have an impact on the current identified trends of temporal structure as was argued in the available research (Abdullahi and Coetzee, 2017). These results show that the time structure in elite badminton (Olympic Games) is a critical highly trained issue for players trying to perform at the highest level.

### Type of Serve and Effectiveness

The results of serve effectiveness showed neutral values when analyzing all the players, medalists and opponents serving (49.6, 49.4, and 50.1%). The current results are in agreement with Bialik (2016) who identified that the serve is not an advantage in badminton. Thus, serving can be considered as a way to start the point that should potentially gain some spatial and temporal (e.g., short, flick, or long serves) advantage over the receiver during the consecutive strokes played in each point (Pearce, 2002; Alcock and Cable, 2009). However, the results showed significant relationships of points won serving for medalists and opponents. Specifically, medalists won more points serving via the backhand flick and backhand short serve; while opponents won more points serving using the forehand flick and forehand short serve. In particular, as the serve is not affected by any previous action of the opponent, the server should manage the most effective serve during each context of badminton matches (Abdullahi and Coetzee, 2017; Chiminazzo et al., 2018). Thus, opponents start the point with less risky serves (e.g., forehand ones) than medalists (Yavad et al., 2007).

### Two-Step Cluster Analysis

Successful actions in badminton require forcing the opponent to perform under spatial and temporal conditions (e.g., close to the net, moving from corner to corner, or corner-net-corner sequences) and then, generating open spaces to win the rally (Chow et al., 2014). Despite this general tactical approach, players have to serve trying to gain some advantage (spatial) during the next strokes to counteract the opponent’s behaviors (Bialik, 2016). Therefore, due to the complex nature of badminton and the neutral serve effectiveness (i.e., ranging from 46 to 56%) the use of different type of serves during matches may allow successful players to adapt to the different scenarios that they have to deal with. In particular, the results of the two-step cluster analysis showed how successful players used the serve when playing short rallies with the backhand short and flick serves (cluster 1: 6.27 s, during sets 1 and 2 and 49.5% of serve effectiveness), and forehand long serves (cluster 2: 7.87 s, during set 2 and 44.7% of serve effectiveness); and during longer rallies with the use of the backhand short serve, forehand short serve and forehand long serve (cluster 3: 12.3 s, during sets 2 and 3, and 56.9% of effectiveness). These results reinforce the idea that successful players are better prepared technically to execute a wide variety of serves according to each specific context (Taylor et al., 2008). In particular, the analysis of quick rallies such as clusters 1 and 2 reflected a lower serving effectiveness (49.5 and 44.7%, respectively) during sets 1 and 2. However, the results of cluster 3 may point to the better tactical and mental preparation to be more successful (56.9% of serve effectiveness) during long rallies played mainly during sets 2 and 3. This performance is related to successful players in elite badminton that can adapt the intensity required during the rallies using a variety of tactical patterns that lead to more successful actions as the match goes on (Barreira et al., 2016; Chiminazzo et al., 2018). In addition, these actions (cluster 3) lead to long rest times (ranging from 21 to 37 s) for medalists to manage fatigue and pressure before serving the next point.

On the other hand, medalists and their opponents played the rallies in a similar way using forehand long serves during set 1 with durations of 8.80 s and 49.0% of effectiveness (cluster 5). In particular, after the introduction of the new scoring system the forehand long serve is the most used serve to start the point and to generate cross-court shots forcing the opponent to run and return the shuttlecock (Alcock and Cable, 2009). Therefore, this result may suggest that at the beginning of the match both players perform with this serve trying to disrupt the opponent’s strategies as a way to induce fatigue and constant adaptation to each stroke (Chow et al., 2014).

Lastly, the opponents showed an independent performance using the forehand short serve during sets 1 and 2, with short rally times (6.63 s) and neutral effectiveness (51%). As was identified in previous studies (Laffaye et al., 2015; Chiminazzo et al., 2018) the performance required at the highest level may lead competitors to play below or beyond the competition requirements, and then opponents may be forced to play taking less risks when serving via short serves. Additionally, when medalists are receiving, they showed better strategical preparation against forehand serves (probably more predictable serves for them) and then performed anticipatory actions forcing the opponent to play quick rallies that involve 3 to 5 strokes (Yadav et al., 2007; Chiminazzo et al., 2018).

### Classification Tree Analysis

The results of the classification tree analysis identified the importance of different serving patterns for each medalist based on some key variables (type of serve, round, rally time, and set) to classify their performances from a multivariate (integrated) approach. Particularly, the gold medalist was characterized by the use of more backhand and forehand flick serves, and the dominant use of the backhand short serve during sets 1 and 2 in all the tournament stages. On the contrary, the bronze medalist showed more forehand long serves during all sets. Lastly, the silver medalist showed a mixed performance of serves using the forehand short serve, the backhand short serve during set 3 of the group stage and round of 16, and the forehand long serve during the final and semi-finals. These findings reflect the importance of identifying individual playing patterns (profiles) that help players to be aware of serving strategies and performance areas they need to monitor according to the opponent (O’Donoghue, 2013). In particular, the silver medalist is a versatile player than manages a wider range of serve types than the gold and bronze medalists; and the bronze medalist mainly used forehand long serves. Therefore, these individual performance profiles based on multiple performance indicators make it possible to describe the idiosyncratic playing patterns of each player from technical, tactical, and strategical approaches (O’Donoghue, 2013). Thus, the analysis of successful players (medalists) is needed in order to update and manage the current player’s performance and its evolution tournament to tournament in accordance with the serving and playing patterns identified (Menescardi et al., 2019).

The present study has some limitations that need to be acknowledged and addressed in future research. On the one hand, it analyzed the matches played by medalists but the opponents playing patterns were neither identified nor monitored during the multivariate analysis (classification and decision tree). As was identified, the type of rallies and playing patterns should be studied in depth in order to analyze the different players’ profiles in elite men and women’s badminton from a long-term perspective (i.e., individual players and by country/continent). On the other hand, further studies should consider larger datasets (Super Series, European and World Championships) to test the importance of winning/losing and successful/unsuccessful conditions. Additionally, some variables can be included to analyze the ending action (type of technique), the zones of the court or the sequence of actions in each rally due to the importance of dynamic analysis in racket sports.

The findings of the current study have some practical implications that can be implemented during training and competitions. The information obtained about the temporal-related demands during matches played by successful players can be used to simulate these competitive scenarios during high-intensity sets/matches. Specifically, identified trends obtained with the two-step cluster analysis can help coaches and players to monitor different serving and playing patterns during training and matches according to the serve and rally requirements. Lastly, the analysis of each medalist’s performance profile makes it possible to better describe and identify the serving strategies during tournaments. This individual approach would allow anticipating how to take advantage (i.e., most used strokes and tactics) of successful players according to serving strategies and playing patterns.

## Data Availability Statement

The datasets generated for this study are available on request to the corresponding author.

## Ethics Statement

Written informed consent was not required, as the data used was publicly available on championship TV.

## Author Contributions

All co-authors equally contributed to the manuscript. M-ÁG-R designed, wrote, and supervised the draft of the article. AC collected the data and described the main variables and factors to use for. FR collected the data, discussed the design, and analyzed the article. L-MR contributed to the introduction, rationale and discussion of main findings, and supervised the final draft of the document.

## Conflict of Interest

FR was employed by company Spanish Badminton Federation. The remaining authors declare that the research was conducted in the absence of any commercial or financial relationships that could be construed as a potential conflict of interest.
